# Supplementary material for: Perturbation of Circadian Rhythm Is Associated with Increased Prevalence of Chronic Kidney Disease: Results of the Korean Nationwide Population-Based Survey
Source: Int J Environ Res Public Health. 2022 May 8;19(9):5732. doi: 10.3390/ijerph19095732 (PMC9102791; doi:10.3390/ijerph19095732)
Supplement: Supplementary file 1 [file ijerph-19-05732-s001.zip › ijerph-1664696-supplementary.pdf]

International Journal of
Environmental Research and Public Health

MDPI

Supplementary Materials

Table S1. General characteristics of participants.

	Control (N=16,779)		CKD (N=629)		p
	N (or MEAN)	% (or STD)	N (or MEAN)	% (or STD)	
SEX					<.0001
Male	7242	43.16	351	55.8	
Female	9537	56.84	278	44.20	
Age	50.97	16.70	60.11	16.79	<.0001
BMI	23.92	3.53	25.09	3.95	<.0001
Education					<.0001
Completion of middle school	5030	30.02	308	48.97	
Higher than high school	11728	69.98	321	51.03	
Smoking status					<.0001
No	10185	60.85	306	48.88	
Yes	6553	39.15	320	51.12	
Drinking status					<.0001
No	1825	10.90	105	16.75	
Yes	14918	89.10	522	83.25	
Physical activity					<.0001
<2 days/week	15404	91.83	607	96.50	
≥2 days/week	1371	8.17	22	3.50	
Diabetes					<.0001
No	15305	91.22	425	67.57	
Yes	1474	8.78	204	32.43	
Hypertension					<.0001
No	12876	76.74	262	41.65	
Yes	3903	23.26	367	58.35	
Sleep onset time					<.0001
Early bedtime	1990	11.98	144	22.97	
Mid bedtime	13261	79.81	443	70.65	
Late bedtime	1365	8.21	40	6.38	
Sleep duration					0.004
<6 hours/day	2389	14.24	76	12.08	
6–<8 hours/day	9319	55.54	325	51.67	
≥8 hours/day	5071	30.22	228	36.25	
SBP	118.54	16.54	127.72	19.12	<.0001
DBP	75.35	10.05	76.05	13.19	0.189
Cr	0.8	0.17	1.28	1.00	<.0001
FBG	100.7	23.24	117.47	43.79	<.0001
TG	134.64	109.49	170.98	149.65	<.0001

The data are shown as N (%) for categorical variables or mean for continuous variables, and p-values were calculated using the chi-squared test or Student's t-test. CKD, chronic kidney disease (CKD was defined by eGFR threshold of 75, 60, and 45 mL/min/1.73 m^2 for younger than 40, 40 to 64, and 65 years or older, respectively, or a positive dipstick urinalysis); N, number; STD, standard deviation; BMI, body mass index; SBP, systolic blood pressure; DBP, diastolic blood pressure; Cr, creatinine; FBG, fasting blood glucose; TG, triglyceride.

Table S2. Associations between sleep onset time and CKD in stratified analyses.

	Early bedtime				Mid bedtime	Late bedtime				P for Interaction
	OR	95% CI		*p*		OR	95% CI		*p*	
Age										0.229
<65	1.197	0.810	1.768	0.367	Ref	1.082	0.723	1.619	0.701	
>=65	1.051	0.867	1.273	0.615	Ref	1.789	1.091	2.933	0.021	
SEX										0.637
Male	1.011	0.800	1.278	0.924	Ref	1.295	0.836	2.006	0.247	
Female	1.092	0.855	1.396	0.479	Ref	1.713	1.103	2.661	0.017	
BMI										0.453
<25	1.106	0.883	1.384	0.382	Ref	1.727	1.156	2.580	0.008	
>=25	0.981	0.759	1.269	0.886	Ref	1.203	0.738	1.962	0.459	
Education										0.878
Completion of middle school	1.064	0.871	1.300	0.544	Ref	1.629	0.990	2.680	0.055	
Higher than high school	1.019	0.728	1.426	0.912	Ref	1.393	0.935	2.077	0.103	
Smoking status										0.944
No	1.044	0.830	1.311	0.715	Ref	1.561	0.993	2.454	0.054	
Yes	1.057	0.822	1.359	0.667	Ref	1.413	0.922	2.164	0.113	
Drinking status										0.643
No	1.090	0.778	1.527	0.615	Ref	2.066	0.966	4.417	0.061	
Yes	1.039	0.852	1.267	0.707	Ref	1.391	0.987	1.960	0.060	
Physical activity										0.150
<2 days/week	1.032	0.867	1.229	0.721	Ref	1.406	1.017	1.943	0.039	
⩾2 days/week	2.570	0.797	8.290	0.114	Ref	3.347	1.084	10.334	0.036	

Adjusted for age, sex, education level, smoking, drinking, physical activity, BMI, SBP, DBP, FBG, TG, comorbidities, such as hypertension, diabetes, hyperlipidemia, cerebrovascular diseases, myocardial infarction, and angina pectoris; OR, odds ratio; CI, confidence interval; Ref, reference.

Table S3. Newly diagnosed CKD prevalence among participants with CKD risk factors or a history of CKD according to sleep onset time.

	MODEL 1				MODEL 2				MODEL 4			
	OR	95% CI		*p*	OR	95% CI		*p*	OR	95% CI		*p*
Sleep onset time				<.0001				0.243				0.303
Early bedtime	1.486	1.249	1.768		0.980	0.817	1.176		0.974	0.808	1.173	
Mid bedtime		REF				REF				REF		
Late bedtime	1.066	0.720	1.578		1.405	0.935	2.113		1.369	0.904	2.075	

Diabetes, hypertension, hyperlipidemia, and cardiovascular disease were identified as risk factors for CKD. Model 1: unadjusted; Model 2: adjusted by age and sex; Model 4: Model 2 + education level, smoking, drinking, physical activity, and BMI; OR, odds ratio; CI, confidence interval; Ref, reference.

Table S4. General characteristics of participants providing work schedule information.

	Control (N=11,125)		CKD (N=431)		*p*
	N (or MEAN)	% (or STD)	N (or MEAN)	% (or STD)	
Sleep onset time					<.0001
Early bedtime	1013	9.21	95	22.14	
Mid bedtime	8996	81.82	312	72.73	
Late bedtime	986	8.97	22	5.13	
Sleep duration					0.003
<6 hours/day	1520	13.66	50	11.6	
6–<8 hours/day	6543	58.81	230	53.36	
≥8 hours/day	3062	27.52	151	35.03	
Work schedule					0.326
Day shift	9608	86.36	387	89.79	
Afternoon shift	1077	9.68	29	6.73	
Night shift	220	1.98	8	1.86	
Split shift	91	0.82	3	0.70	
24-hour rotating shift	129	1.16	4	0.93	

The data are shown as N (%) for categorical variables or mean for continuous variables, and p-values were calculated using the chi-squared test or Student's t-test. CKD, chronic kidney disease; N, number.

Table S5. Newly diagnosed CKD prevalence according to sleep onset time among participants providing work schedule information.

	**MODEL 1**				**MODEL 5**				**MODEL 6**			
	**OR**	**95% CI**		***p***	**OR**	**95% CI**		***p***	**OR**	**95% CI**		***p***
Sleep onset time				0.018				0.105				0.329
Early bedtime	2.160	1.266	3.685		1.859	1.045	3.306		1.524	0.821	2.828	
Mid bedtime		REF				REF				REF		
Late bedtime	1.191	0.700	2.025		1.098	0.616	1.960		1.232	0.674	2.252	

CKD was defined as new onset CKD in participants without CKD risk factors or a history of CKD. Model 1: unadjusted; Model 5: adjusted by age, sex, work schedule; Model 6: Model 5 + education level, smoking, drinking, physical activity, BMI and sleep duration; OR, odds ratio; CI, confidence interval; Ref, reference.
